# α‐Motor neurons are spared from aging while their synaptic inputs degenerate in monkeys and mice

**DOI:** 10.1111/acel.12726

**Published:** 2018-02-04

**Authors:** Nicholas Maxwell, Ryan W. Castro, Natalia M. Sutherland, Kelli L. Vaughan, Mark D. Szarowicz, Rafael de Cabo, Julie A. Mattison, Gregorio Valdez

**Affiliations:** ^1^ Virginia Tech Carilion Research Institute Virginia Tech Roanoke VA USA; ^2^ Graduate Program in Translational Biology, Medicine, and Health Virginia Tech Blacksburg VA USA; ^3^ Translational Gerontology Branch National Institute on Aging NIH Baltimore MD USA; ^4^ SoBran, Inc. Burtonsville MD USA; ^5^ Department of Biological Sciences Virginia Tech Blacksburg VA USA

**Keywords:** aging, alpha‐motor neuron, synapse, lipofuscin, neurodegeneration, spinal cord

## Abstract

Motor function deteriorates with advancing age, increasing the risk of adverse health outcomes. While it is well established that skeletal muscles and neuromuscular junctions (NMJs) degenerate with increasing age, the effect of aging on α‐motor neurons and their innervating synaptic inputs remains largely unknown. In this study, we examined the soma of α‐motor neurons and innervating synaptic inputs in the spinal cord of aged rhesus monkeys and mice, two species with vastly different lifespans. We found that, in both species, α‐motor neurons retain their soma size despite an accumulation of large amounts of cellular waste or lipofuscin. Interestingly, the lipofuscin profile varied considerably, indicating that α‐motor neurons age at different rates. Although the rate of aging varies, α‐motor neurons do not atrophy in old age. In fact, there is no difference in the number of motor axons populating ventral roots in old mice compared to adult mice. Moreover, the transcripts and proteins associated with α‐motor neurons do not decrease in the spinal cord of old mice. However, in aged rhesus monkeys and mice, there were fewer cholinergic and glutamatergic synaptic inputs directly abutting α‐motor neurons, evidence that aging causes α‐motor neurons to shed synaptic inputs. Thus, the loss of synaptic inputs may contribute to age‐related dysfunction of α‐motor neurons. These findings broaden our understanding of the degeneration of the somatic motor system that precipitates motor dysfunction with advancing age.

## 1 INTRODUCTION

Motor function progressively erodes as individuals transition from adulthood into old age. Aging compromises gait speed, balance, and the command of fine motor skills (Fried, Ferrucci, Darer, Williamson & Anderson, 2004; Sorond et al., 2015), increasing the risk of injury due to falls and age‐associated diseases (Ambrose, Paul & Hausdorff, 2013; Bennett et al., 1996; Camicioli, Moore, Sexton, Howieson & Kaye, 1999; Martin, 2011; Richards, Stern & Mayeux, 1993; Soriano, DeCherrie & Thomas, 2007; Verghese, Wang, Lipton, Holtzer & Xue, 2007). Age‐ and disease‐related motor deficits occur because of deleterious changes in neuronal and non‐neuronal components of the somatic nervous system (Bennett et al., 1996; Boillée et al., 2006; Dentel et al., 2013; Fiatarone & Evans, 1993). For example, we recently showed that IA/II proprioceptive sensory neuron soma and nerve ending at muscle spindles degenerate with increasing age and progression of amyotrophic lateral sclerosis (ALS) in mice (Vaughan, Kemp, Hatzipetros, Vieira & Valdez, 2015; Vaughan, Stanley & Valdez, 2016). These sensory neurons detect changes in the amount and rate of muscle contraction, and utilize this information to coordinate and modulate the activity of α‐motor neurons. Hence, their degeneration would inevitably compromise motor function.

In addition, aging has been linked to deleterious physiological alterations. For example, it is well established that, with increasing age, extrafusal muscle fibers atrophy (Hepple, 2003; Marzetti et al., 2012; McKenzie, Bua, McKiernan, Cao & Aiken, 2002) and neuromuscular junctions (NMJs) degenerate (Fahim, 1997; Kelly & Robbins, 1983; Tintignac, Brenner & Rüegg, 2015; Valdez et al., 2010). Moreover, aging causes cellular and physiological alterations along innervating motor axons (Apel et al., 2009; Kanda & Hashizume, 1989; Kang & Lichtman, 2013) and alters the expression of genes with critical functions at NMJs, and elsewhere in skeletal muscles (Jang, Sinha, Cerletti, Dall'Osso & Wagers, 2011; McKenzie et al., 2002; Nishimune et al., 2012; Weisleder et al., 2006).

Although α‐motor neurons receive, integrate, and relay all motor commands to skeletal muscles, the effect of aging on these neurons is still debated. Several studies, using histological assays to sample spinal cord sections, have reported that fewer α‐motor neurons are present in aged humans and animals (Jacob, 1998; Tomlinson & Irving, 1977). However, these same studies show that the remaining α‐motor neurons enlarge rather than atrophy with age. Yet, other studies have found no difference in the number and size of α‐motor neurons in old compared to adult mice (Chai, Vukovic, Dunlop, Grounds & Shavlakadze, 2011). Thus, the effect of aging on α‐motor neurons is still an open question, and addressing it will contribute significantly to our understanding of NMJ and muscle fiber degeneration and accompanying age‐related motor deficits.

There is even less known about the fate of synaptic inputs in aging spinal cords, which are responsible for initiating and modulating all voluntary movements. Synaptic inputs terminate throughout the soma and dendrites of α‐motor neurons (Witts, Zagoraiou & Miles, 2014) and include excitatory (glutamatergic and cholinergic) and inhibitory (GABAergic and glycinergic) inputs originating from a variety of neurons. Glutamatergic inputs are largely responsible for activating α‐motor neurons and emanate from neurons located throughout the central and peripheral nervous system. Cholinergic inputs, called C‐boutons, modulate the output of α‐motor neurons and arise from small interneurons located near the midline of the spinal cord. GABAergic and glycinergic inhibitory inputs originate from interneurons located in the spinal cord and are critical for fine‐tuning and terminating the activity of α‐motor neurons. Although these inputs have yet to be examined in the context of aging, they have been shown to degenerate in several diseases known to affect motor function, including ALS (Vaughan et al., 2015). Thus, age‐associated dysfunction or loss of synaptic inputs terminating on α‐motor neurons will also impair motor function.

In this study, we carried out histological and molecular analysis to determine the impact of aging on α‐motor neurons and cholinergic inputs in the spinal cord of rhesus monkeys and mice. We examined these two species for several reasons: (i) Their vastly different average lifespans (25 years for rhesus monkeys and 2.12 years for C57/BL6 mice) make it possible to determine whether cellular changes are due to biological or chronological aging, (ii) rhesus monkeys are an important animal model for understanding human aging because they share approximately 93% of their genetic code with humans, and (iii) mice provide a tractable model system for interrogating age‐related changes due to their small size and relatively short lifespan. In both species, we used light microscopy to examine the size of α‐motor neuron somata, the presence of lipofuscin, the number of motor axons, and the glutamatergic and cholinergic inputs on α‐motor neuron somata and throughout the ventral horn. In addition, molecular analyses were conducted for transcripts and proteins associated with motor neurons.

## 2 RESULTS

### 2.1 α‐Motor neurons do not atrophy in old rhesus monkeys

We first examined the morphology of α‐motor neurons in the spinal cord of rhesus monkeys and mice. To visualize α‐motor neurons, 30‐μm spinal cord cross sections were stained with an antibody against the pan‐neuronal marker, Neuronal Nuclei (NeuN) (Mullen, Buck & Smith, 1992), and an antibody against the vesicular acetylcholine transporter (VAChT). We identified α‐motor neurons based on: (i) their location in the ventral horn of the spinal cord, (ii) the large size of α‐motor neurons compared to other neurons in the spinal cord, and (iii) the presence of VAChT puncta, cholinergic inputs called C‐boutons, throughout the perimeter of the soma and along the apical dendrite of α‐motor neurons.

**Figure 1:**
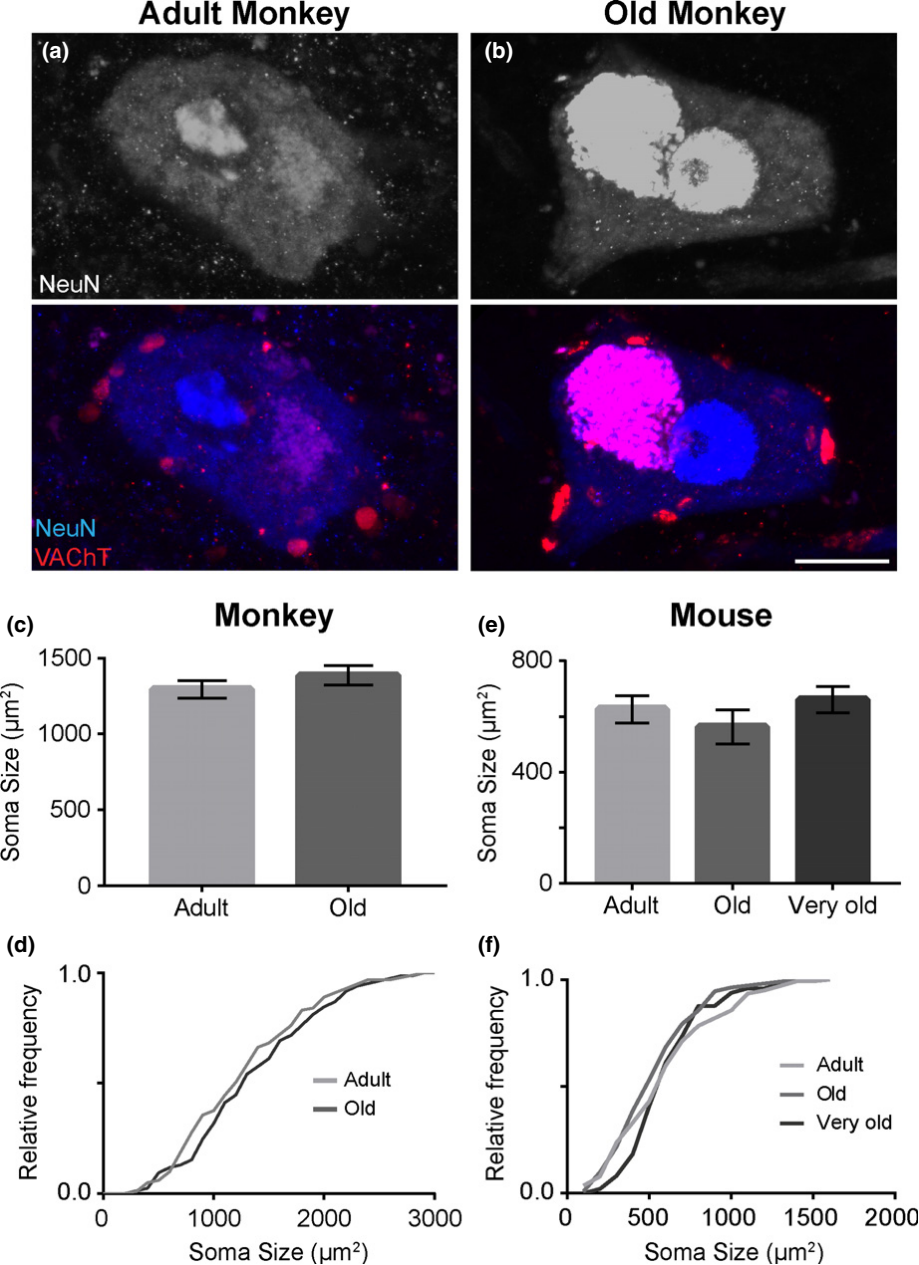
The soma size of α‐motor neurons remains unchanged in aged rhesus monkeys and mice. α‐Motor neurons were identified using antibodies against NeuN (Blue) and VAChT (Red) (a, b). There is no difference in the average and frequency distribution of soma size between old and adult rhesus monkeys (c, d). The same analysis showed that the size of α‐motor neurons is unchanged in the lumbar region of adult, old, and very old mice (e‐f). Error bar = standard error. Scale bar = 20 μm

First, we compared the soma size between adult and old male rhesus monkeys. As depicted in Table 1, we analyzed the size of α‐motor neurons in the spinal cord of adult (6‐ to 17‐year‐old) and old (28‐ to 32‐year‐old) monkeys (Figure 1a–b). This analysis revealed no significant difference in the average soma size of old (1,388 ± 102.4 μm^2^) compared to adult (1,231 ± 152.1 μm^2^) rhesus monkeys (Figure 1c). While these findings suggest that α‐motor neurons do not atrophy in old rhesus monkeys, it remained possible that aging affects a subpopulation of α‐motor neurons. For example, aging may preferentially affect slow, fast fatigue‐resistant (FFR) or fast‐fatigable (FF) α‐motor neurons. These neurons vary in size (slow<FFR<FF), functional demands, and susceptibility to diseases (Stifani, 2014). If aging preferentially affects a subtype of α‐motor neurons, the distribution of α‐motor neurons soma size should be different between adult and old animals. However, a frequency histogram and a two‐sample Kolmogorov–Smirnov (KS) test revealed no difference in the soma size distribution between adult and old rhesus monkeys (Figure 1d), indicating that, in rhesus monkeys, α‐motor neurons retain their soma size throughout the aging process.

**Table 1:** Spinal cord samples from male rhesus monkeys

	Age (years)	Cervical	Lumbar	Thoracic	Total
Adult	6–17	7	1	1	9
Old	28–32	3	4	3	10

### 2.2 α‐Motor neurons do not atrophy in old mice

We next assessed the morphology of α‐motor neurons in the spinal cord of C57BL/6 adult (2‐ to 3‐month‐old), old (21‐month‐old), and very old (28‐month‐old) male mice (*N* = 4 per age group for this and all other experiments involving mice). To this end, α‐motor neurons residing in the lumbar region of the spinal cord were examined. To identify α‐motor neurons, 30‐μm spinal cord cross sections were stained with antibodies against NeuN and VAChT. As in rhesus monkeys, the average size of α‐motor neurons remains unchanged in old and very old mice compared to adult mice (adult = 626.9 ± 49.03 μm^2^; old = 562.7 ± 61.95 μm^2^: *p*‐value = .4434; very old mice = 660.9 ± 47.27 μm^2^: *p*‐value = .6916; Figure 1e). A frequency histogram and a two‐sample KS test also showed that aging does not preferentially affect a subset of α‐motor neurons, because the soma size distribution is similar between adult, old, and very old mice (adult vs. old: *p* = .2120; adult vs. very old: *p* = .1098; Figure 1f). We extended this analysis to α‐motor neurons located in the thoracic and cervical regions of the spinal cord. As in the lumbar region, the size of α‐motor neurons remains unchanged in the thoracic and cervical regions in old and very old mice (Figure S1).

**Figure 2:**
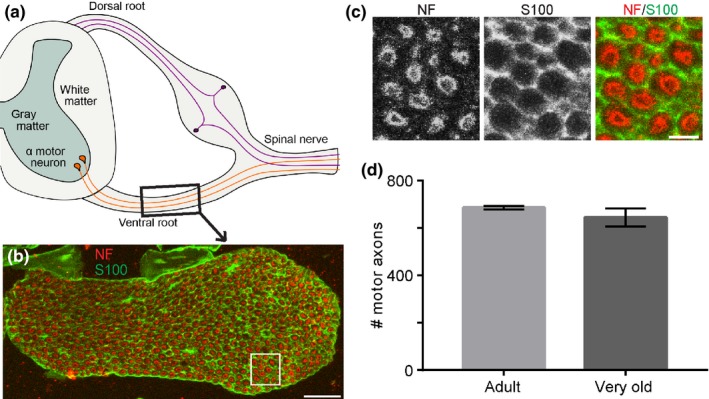
The number of motor axons close to the spinal cord does not decrease with advancing age in mice. Analysis of motor axons in L3 ventral roots in mice (a). Ventral roots were stained with antibodies against neurofilament to visualize axons (Red) and S100 to label Schwann cells (Green) (b, c). There is no difference in the number of motor axons between adult and very old mice (d). Error bar = standard error. Scale bar = 100 μm (b) and 10 μm (c)

In addition, we counted the number of motor axons in L3 ventral roots in adult and very old mice (Figure 2a). Axons were visualized in 15‐μm cross sections using an antibody against neurofilament. The same tissue sections were also stained with an antibody against S100, a Schwann cell marker (Figure 2b,c). This analysis revealed no difference in the number of axons in L3 ventral roots between adult and very old mice (adult = 685.6 ± 7.232; very old = 644.4 ± 38.14: *p*‐value = .2675; Figure 2d). Taken together, these data demonstrate that, in mice, α‐motor neurons do not degenerate with age.

### 2.3 Aging does not reduce genes preferentially associated with α‐motor neurons in mice

To more broadly assess the effect of aging on α‐motor neurons, we analyzed levels of the homeobox gene 9 (Hb9), the gene encoding for the LIM homeodomain protein 1 (Isl‐1), the choline acetyltransferase (ChAT), and the vesicular acetylcholine transporter (VAChT; Lu, Niu & Alaynick, 2015). These genes play important roles in the differentiation and function of α‐motor neurons. Their expression patterns thus correlate with the number and functional status of α‐motor neurons. Using qPCR, we found mRNA levels for HB9, Isl‐1, ChAT, and VAChT unchanged in the spinal cord of 27‐month‐old mice compared to 4‐month‐old mice (Figure S2a). We also examined protein levels for VAChT and Isl‐1 by Western blotting. As expected, VAChT protein is present at similar levels in the spinal cord of very old and adult mice (Figure S2b,c). We then used an antibody that detects Isl‐1 and Isl‐2 to assess changes in the aged mice. This antibody showed that Isl‐1 and Isl‐2 proteins significantly increase in very old mice (Figure S2d,e). It also revealed that levels of Isl‐1 mRNA and protein levels are uncorrelated in the spinal cord of very old mice. Additionally, we examined levels of NeuN to explore the effect of aging on most spinal cord resident neurons (Figure S3). Like Isl‐1, we found that, while aging did not alter the abundance of NeuN transcripts (Figure S3a), it significantly increased levels of NeuN proteins in the spinal cord of very old mice (Figure S3b,c). Together, these findings show that genes associated with motor neurons are either unchanged or increased in very old age. These findings further indicate that the number of α‐motor neurons is unchanged in aged animals.

### 2.4 Lipofuscin accumulation reveals that motor neurons age at different rates within and between animals

During this study, we found that lipofuscin progressively aggregates in the perinuclear region of aging α‐motor neurons of mice and monkeys (Figure S4). Lipofuscin, a form of cellular waste, preferentially accumulates in the cytosol of large neurons in animals and humans as they age (Gray & Woulfe, 2005; Keller et al., 2004). Lipofuscin is composed of lipids and highly oxidized and cross‐linked proteins that accumulate within lysosomes. Because lipofuscin auto‐fluoresces, it is easy to separate from proteins and subcellular structures labeled with specific fluorophores using light microscopy. In old rhesus monkeys, the number of α‐motor neurons with aggregated lipofuscin was significantly higher compared to adult monkeys (adult = 44.57 ± 4.698%; old = 78.1 ± 5.021%; *p* < .0001; Figure S4a–c). In mice, the incidence of α‐motor neurons with aggregated lipofuscin increased with advancing age (adult = 0%; old = 73 ± 9.891%; very old = 87.33 ± 3.844%; Figure S4d). These findings were not surprising given that α‐motor neurons are relatively large compared to other neurons, and lipofuscin has been primarily found in large neurons (Liang, Nelson, Yazdani, Pasbakhsh & German, 2004). As the soma size and number of α‐motor neurons appear unchanged in old age, lipofuscin likely marks other well‐documented aged‐related afflictions known to affect α‐motor neurons, such as degeneration of motor nerve endings at NMJs.

We made two additional and notable observations while analyzing lipofuscin in both rhesus monkeys and mice. First, we found aged α‐motor neurons with vastly different lipofuscin profiles (Figure S4a,b) often adjacent to each other and within the same spinal cord segment. In both aged monkeys and mice, lipofuscin aggregates were either absent, or, in small, or large amounts in these neurons. These varied lipofuscin profiles indicate that α‐motor neurons age at different rates. Second, we found that lipofuscin begins to aggregate in α‐motor neurons of adult rhesus monkeys (Figure S4c). In stark contrast, lipofuscin aggregates are absent from α‐motor neurons of adult mice (Figure S4d). We also found significantly more α‐motor neurons with lipofuscin aggregates and occupying more of the cytosol in old rhesus monkeys (Figure S4e) compared to old mice (Figure S4f). This interspecies comparison suggests that the accumulation of lipofuscin correlates with chronological rather than biological aging as lipofuscin is found accumulated in α‐motor neurons of adult rhesus monkey but not adult mice (Figure S4c–f).

### 2.5 Cholinergic inputs decrease in the spinal cord of aged rhesus monkeys and mice

**Figure 3:**
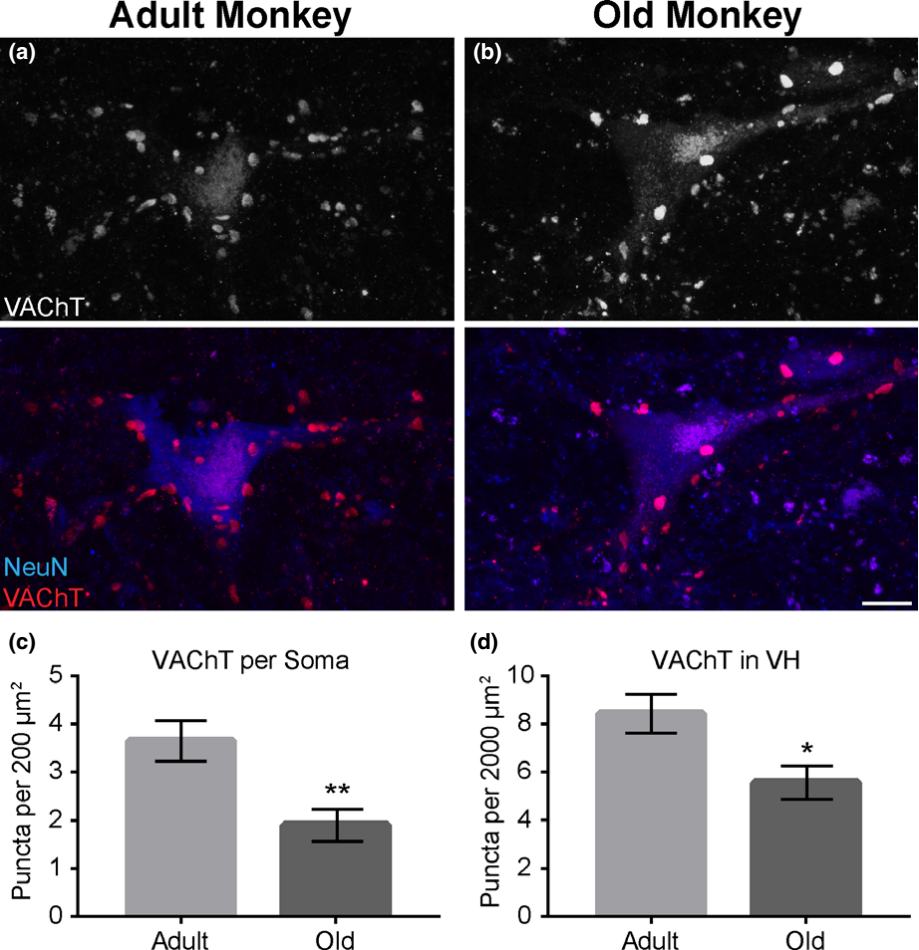
The number of C‐boutons innervating motor neurons decreases in old rhesus monkeys. C‐boutons were visualized with anti‐VAChT (Red) and the motor soma with anti‐NeuN (Blue) (a, b). In old rhesus monkeys, fewer C‐boutons are present on the motor soma (c). Aging also reduces the total number of C‐boutons in the ventral horn (d). Error bar = standard error. *p ≤* *.05. **p ≤* *.01. Scale bar = 20 μm

As synapses have been shown to degenerate early, progressively, and prior to soma atrophy that occurs with increasing age (Valdez et al., 2010), we asked if aging affects synaptic inputs critical for the function of α‐motor neurons. With immunostaining for VAChT, we visualized cholinergic inputs, C‐boutons, terminating on α‐motor neurons. In old rhesus monkeys, there were fewer C‐boutons specifically terminating on α‐motor neuron somata compared to adults (adult = 3.646 ± 0.4229; old = 1.899 ± 0.3367 *p*‐value = .006; Figure 3a–c). The total number of C‐boutons in the ventral horn was also reduced in old rhesus monkeys (adult = 8.427 ± 0.8021; old = 5.557 ± 2.212 *p*‐value = .0173; Figure 3d).

**Figure 4:**
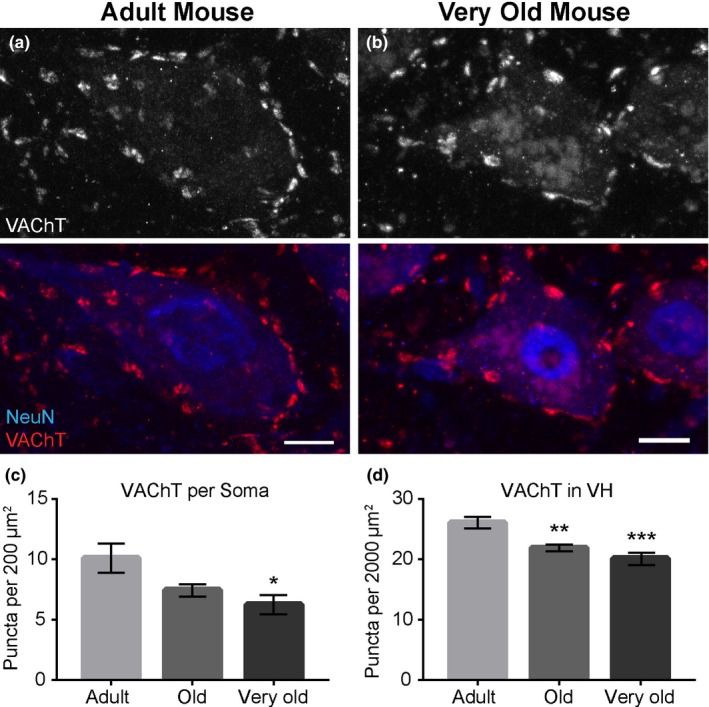
In mice, the number of C‐boutons innervating motor neurons decreases with advancing aging. C‐boutons were visualized anti‐VAChT (Red) and the motor soma with anti‐NeuN in the lumbar region of the spinal cord (a, b). Fewer C‐boutons are present on the motor soma of old and very old mice compared to adult mice (c). Aging also reduces the total number of C‐boutons in the ventral horn of mice (d). Error bar = standard error. *p ≤* *.05. **p ≤* *.01. ***p ≤* *.001. Scale bar = 20 μm

In very old mice, there were fewer C‐boutons located specifically on the soma of α‐motor neurons of the lumbar region compared to adult mice (adult = 10.12 ± 1.218; old = 7.453 ± 0.503: *p*‐value = .0790; very old = 6.263 ± 0.8037: *p*‐value = .0301, compared to adult; Figure 4a–c). The total number of C‐boutons was also reduced in the ventral horn of old and very old mice compared to adult mice (adult = 26.07 ± 0.9389; old = 21.92 ± 0.5429: *p*‐value = .0011; very old = 20.13 ± 1.011: *p*‐value = .0002; Figure 4d). To determine whether C‐boutons degenerate in other regions of the spinal cord with advancing age, we carried out the same analysis in the cervical and thoracic regions. Similar to the lumbar region, there were fewer C‐boutons on the somata of α‐motor neurons and throughout the ventral horn of the cervical and thoracic regions of the spinal cord (Figure S5). These findings indicate that C‐boutons degenerate in the spinal cord of aged rhesus monkeys and mice. As VAChT protein is unchanged in the spinal cord of very old mice, the loss of VAChT‐positive puncta, C‐boutons, may result from a reduced level of VAChT at synaptic sites with a concomitant increase elsewhere along axons or the soma of small cholinergic interneurons and α‐motor neurons.

**Figure 5:**
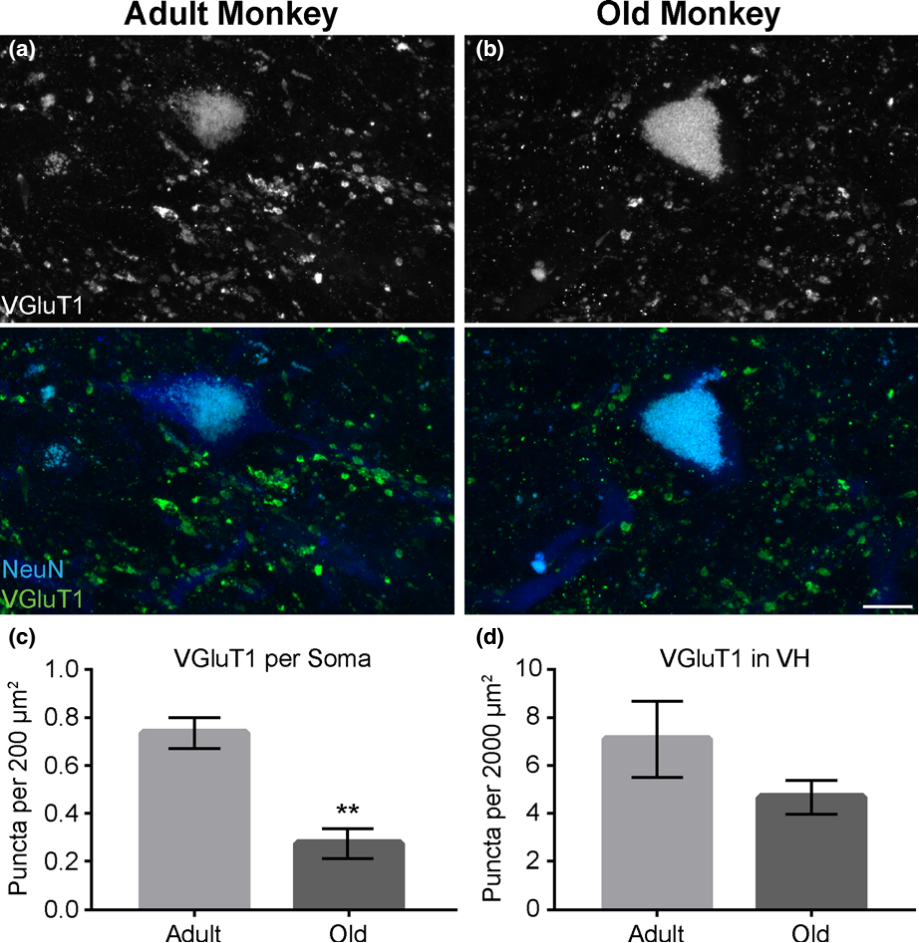
VGLUT1‐positive synaptic inputs innervating motor neurons decrease in old rhesus monkeys. Immunostaining for VGLUT1 (Green) and the motor soma with anti‐NeuN (Blue) (a, b). Fewer VGLUT1 puncta are present on the motor soma of old compared to adult rhesus monkeys (c). The total number of VGLUT1 puncta is also reduced in the ventral horn of rhesus monkey (d). Error bar = standard error. **p ≤* *.01. Scale bar = 20 μm

### 2.6 Glutamatergic inputs terminating on the somata of α‐motor neurons decrease in the spinal cord of aged rhesus monkeys and mice

Additionally, we examined the impact of aging on glutamatergic inputs. Glutamatergic inputs were visualized by staining 30‐μm spinal cord sections with an antibody against the vesicular glutamate transporter 1 (VGLUT1). While VGLUT1‐positive inputs were present in all regions of the spinal cord, only VGLUT1‐positive inputs in the ventral horn, where they directly innervate α‐motor neurons and thus modulate somatic motor function, were examined. These VGLUT1 inputs were significantly reduced in old rhesus monkeys compared to adult (adult = 0.7365 ± 0.0645; old = 0.274 ± 0.0631; *p*‐value = .0015; Figure 5a–c). However, the total number of VGLUT1 inputs in the ventral horn was not significantly decreased in old rhesus monkeys (adult = 7.096 ± 1.584; old = 4.674 ± 0.7017; *p*‐value = .1514; Figure 5d).

**Figure 6:**
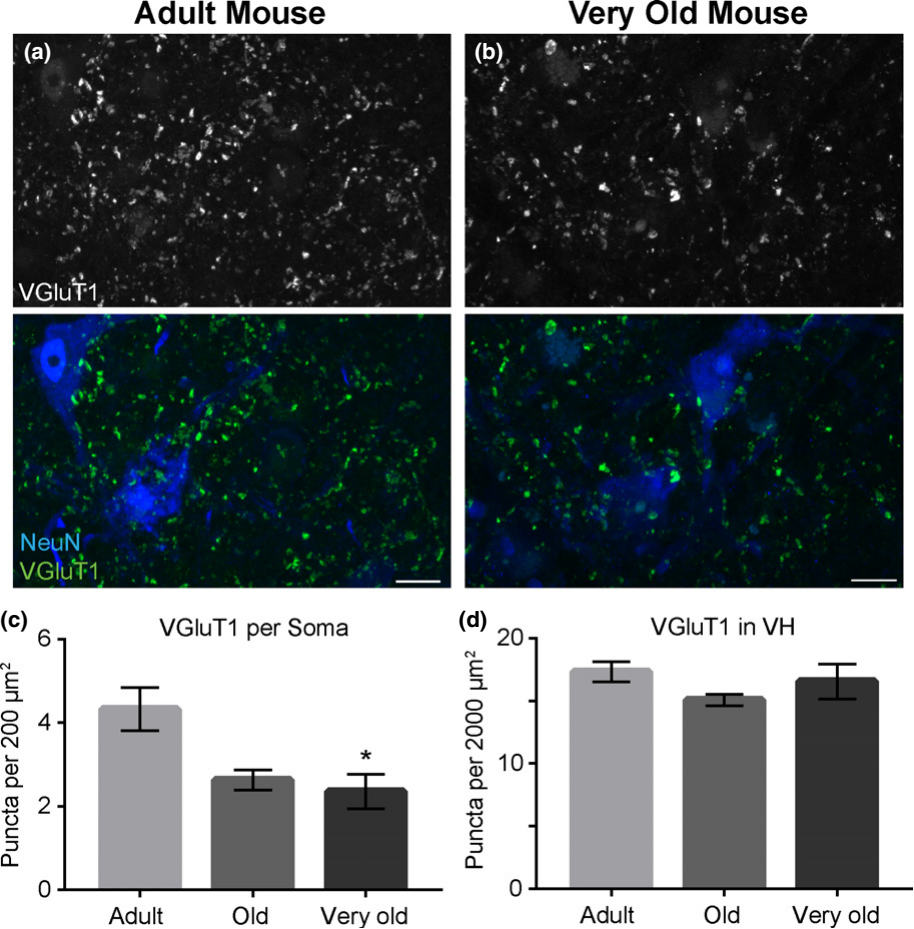
In mice, VGLUT1‐positive synaptic inputs innervating motor neurons decrease with advancing age. Immunostaining for VGLUT1 (Green) and the motor soma with anti‐NeuN (Blue) in the lumbar region of the spinal cord (a, b). There are significantly fewer VGLUT1 puncta present on the motor soma of very old mice compared to adult mice (c). However, the total number of VGLUT1 puncta does not change in the ventral horn of mice (d). Error bar = standard error. *p ≤* *.05. Scale bar = 20 μm

In mice, the number of VGLUT1‐positive inputs directly terminating on the somata of α‐motor neurons also decreased with advancing age in the lumbar, thoracic, and cervical regions of the spinal cord (Figure 6a–c and Figure S6a,c). The total number of VGLUT1‐positive inputs throughout the ventral horn, however, is unchanged in the lumbar, thoracic, and cervical regions of the spinal cord of aged mice (Figure 6d, and Figure S6b,d). These findings show that aging contributes to degeneration of glutamatergic synaptic inputs directly terminating on α‐motor neurons in mice.

How is it possible that VGLUT1‐positive inputs on the somata of α‐motor neurons decrease without a concomitant decrease in the entire ventral horn of aged rhesus monkeys and mice? One possibility is that VGLUT1‐positive inputs terminating on the somata of α‐motor neurons comprise a rather small portion of those in the entire ventral horn; thus, their loss with age has little impact on the total number in the ventral horn and entire spinal cord. Alternatively, it is plausible that while VGLUT1 inputs on the somata degenerate, axonal nerve endings sprout to form additional VGLUT1‐positive synapses elsewhere in the ventral horn. The formation of new synaptic inputs could make up for the loss of VGLUT1‐positive inputs on the somata. In either of these two scenarios, the level of VGLUT1 protein should remain unchanged in the spinal cord. However, Western blotting revealed a significant increase in VGLUT1 in the spinal cord of aged mice (Figure S7), indicating that aging may instead cause VGLUT1 to accumulate along axons, but away from synaptic inputs. Providing support to this theory, published findings have shown that aging and diseases increase the accumulation of synaptic markers throughout the axoplasm in mice (Valdez, Tapia, Lichtman, Fox & Sanes, 2012).

## 3 DISCUSSION

This report provides strong evidence that aging does not affect the size and number of α‐motor neurons in mice and rhesus monkeys, and α‐motor neurons preserve their soma size even in the presence of high levels of lipofuscin. As evidence that aging does not result in degeneration of α‐motor neurons, we have reported that the number of efferent axons in ventral roots remains unchanged in very old mice. Additionally, the expression of genes associated with cholinergic neurons, including α‐motor neurons, is similar in the spinal cord of aged compared to adult mice. In contrast, cholinergic inputs that modulate the function of α‐motor neurons degenerate in old animals, which supports previously published findings from our laboratory demonstrating that synaptic degeneration preceded neuronal death in the course of normal aging. These findings are significant for understanding age‐related motor deficits in humans because of the highly similar genetic makeup of rhesus monkeys and humans.

### 3.1 Impact of aging on α‐motor neurons

Although several published studies have reported on the morphology and quantity of α‐motor neurons in aged humans and animals, whether α‐motor neurons atrophy or completely degenerate in old age and exactly when these processes occur is still debated. Human studies have revealed that the number of α‐motor neurons precipitously decreases after 60 years of age (Cruz‐Sánchez, Moral, Tolosa, de Belleroche & Rossi, 1998; Kawamura, O'Brien, Okazaki & Dyck, 1977; Tomlinson & Irving, 1977; Zhang, Goto, Suzuki & Ke, 1996). Likewise, fewer α‐motor neurons were found in aged rats (Hashizume, Kanda & Burke, 1988; Jacob, 1998). In stark contrast, other studies found no change in the number of α‐motor neurons in aged cats and mice (Chai et al., 2011; Liu, Bertolotto, Engelhardt & Chase, 1996). There are also contradictory findings on the effect of aging on the size of α‐motor neurons. These discrepancies may be a consequence of the techniques utilized to analyze aged α‐motor neurons. In several studies, α‐motor neurons were identified following staining of nuclei or following the uptake of a fluorescence dye injected into the gastrocnemius muscle and analyzed using low‐resolution microscopy.

In this study, we took a holistic approach to examine the effect of aging on α‐motor neurons in two species with vastly different average and maximum lifespans. We used immunostaining to visualize α‐motor neurons based on their location, size, and innervation of C‐boutons in rhesus monkeys and mice; specifically examined motor axons in the ventral root of aged mice; and assessed transcripts and protein levels of genes associated with α‐motor neurons in aged spinal cords. These complementary approaches indicated that neither the size nor the number of motor neurons decreases in old animals. How can these findings be reconciled with published studies showing that the number of motor axons in humans and mice decreases in old age? In humans, the relationship between axonal loss and cause of death was not established, allowing for the possibility that diseases, rather than normal aging, cause the loss of motor axons. In mice, a previous study (Valdez et al., 2010) used hybrid transgenic mice expressing yellow fluorescence protein (YFP), which has been shown to contribute to degeneration of axons under stress (Bridge et al., 2009), whereas this study examined C57BL/6 mice.

### 3.2 The rate of aging varies among α‐motor neurons in the same anatomical region

We found that α‐motor neurons retain their soma size in the presence of substantial amounts of lipofuscin, demonstrating that lipofuscin is a marker and not a driver of neuronal aging. Interestingly, we identified α‐motor neurons with vastly different lipofuscin profiles. This finding strongly suggests that α‐motor neurons age at different rates, a possibility supported by published data showing that motor axon nerve endings degenerate at different rates, even within the same muscle (Valdez et al., 2012). As the soma size and number of α‐motor neurons appear unchanged in old animals, the aggregation of lipofuscin must correlate with age‐related subcellular changes in α‐motor neurons that include altered neurotransmission (Arasaki, Iwamoto & Tamaki, 1995), deregulated expression of proteins critical for the assembly, and early and progressive degeneration of presynaptic sites at the NMJ (Valdez et al., 2010).

### 3.3 Aging affects cholinergic and glutamatergic synaptic inputs that innervate α‐motor neurons

We show that excitatory inputs, both cholinergic and glutamatergic, degenerate in aged spinal cords despite the lack of atrophy of α‐motor neurons. These findings support published data indicating that synaptic degeneration occurs before connecting cells atrophy in aged animals. In the neocortex of old rhesus monkeys, fewer synapses are found on pyramidal neurons (Morrison & Baxter, 2012). Synapses also degenerate in the hippocampus, cortical regions, and olfactory bulb of aged rodents prior to degeneration of the neuronal soma (Azpurua & Eaton, 2015; Barnes, 1994; Grillo, 2016; Hof & Morrison, 2004; Punga & Ruegg, 2012). The relationship between synaptic and soma changes with aging has been best documented at the NMJ, primarily because of the large size and accessibility of this peripheral synapse. With increasing age, deleterious structural and functional changes occur in the NMJ, culminating in the degeneration of innervating α‐motor nerve endings (Valdez et al., 2010). However, the NMJ degenerates before pathophysiological changes are obvious in muscle fibers and α‐motor neurons. The NMJ and CNS synapses follow a similar fate in diseases associated with aging, such as Alzheimer's, Parkinson's, and ALS. While the underlying reason for the early and progressive degeneration of synapses during normal aging and diseases is under intense investigation, there is growing evidence that aging affects levels and function of target‐derived factors required to maintain and repair synapses. Hence, it is plausible that α‐motor neurons fail to secrete sufficient levels of synaptic organizing factors needed to maintain cholinergic and glutamatergic inputs on their soma and dendrites. Alternatively, retraction of synaptic inputs may be driven by changes within innervating neurons or due to dysfunction of astrocytes. Understanding the factors that cause synaptic degeneration, in both the CNS and periphery, would aid in the development of therapies that preserve motor function into old age.

### 3.4 Implications for understanding age‐related motor dysfunction

In addition to cholinergic inputs, neurons residing within and outside the spinal cord send signals to α‐motor neurons (Lu et al., 2015; Stifani, 2014). Specifically, α‐motor neurons require glutamatergic inputs to function. Hence, future studies will need to determine the effect of aging on the structure and function of glutamatergic, as well as GABAergic and glycinergic, inputs in order to understand the contributing factors that affect the normal function of aging α‐motor neurons and what may lead to age‐related motor deficits. It will also be critical to determine changes in other spinal cord resident cells with an important function in somatic movements, such as gamma‐motor neurons, the role of glial cells in synaptic degeneration, and molecular mechanisms important for preserving synaptic inputs in the spinal cord.

## 4 EXPERIMENTAL PROCEDURES

### 4.1 Mice

Adult (2–4 months), old (21 months), and very old mice (27–28 months) C57BL/6J wild‐type mice were obtained from the National Institute on Aging. Mice were anesthetized using isoflurane and then perfused transcardially with 10 ml of 0.1 m PBS, followed by 25 ml of ice‐cold 4% paraformaldehyde (PFA) in 0.1 m PBS (pH 7.4). Only male mice were analyzed in this study. All experiments were carried out under NIH guidelines and animal protocols approved by the Virginia Tech Institutional Animal Care and Use Committee.

### 4.2 Monkeys

All monkey studies were conducted at the NIH Animal Center under protocols approved by the National Institute on Aging, Intramural Research Program's Animal Care and Use Committee. Subjects were male rhesus monkeys (*Macaca mulatta*), and ages are specified in Table 1. Monkeys were on experimental protocols not specific to the spinal cord collection. Samples were collected following euthanasia for unrelated clinical or experimental purposes; collection occurred within 2 hr of death. Once harvested, samples were immersed in ice‐cold 4% paraformaldehyde (PFA) for 24–48 hr and then kept in cold 0.1 m PBS.

### 4.3 Immunohistochemistry

Mouse and monkey spinal cord sections were blocked using 5% lamb serum, 3% bovine serum albumin, and 0.1% Triton X‐100 in 0.1 m PBS for a minimum of 30 min at room temperature. Sections were then incubated with primary antibodies 72 hr in blocking solution. Following this, sections were washed 3 times with 1× PBS and then incubated for 4–5 hr at room temperature with Alexa Fluor fluorescently tagged secondary antibodies. Sections were then washed 3 times with 0.1 m PBS and then mounted with Vectashield. The primary antibodies and concentrations are displayed in Table 2.

**Table 2:** Primary antibodies used for IHC and western blotting

Antibody	Origin	Isotype	Dilution IHC	Dilution Western blot	Supplier
Anti‐GAPDH	Rabbit	IgG	N/A	1:10,000	Rockland 600‐401‐A33
Anti‐Islet 1/2	Mouse	IgG2b	N/A	1:10,000	DSHB 39.45D
Anti‐NeuN	Mouse	IgG1	1:250	1:10,000	Millipore MAB377
Anti‐VAChT	Guinea Pig	IgG	1:250	N/A	Millipore AB1588
Anti‐VAChT	Rabbit	IgG	N/A	1:1,000	Sigma SAB4200560
Anti‐VGLUT1	Rabbit	IgG	1:1,000	1:1,000	Millipore AB377

### 4.4 Analysis of soma size and synaptic input number

α‐Motor neurons can be identified by their position within the ventral horn of the spinal cord, their relatively large size, and the presence of C‐boutons on the soma. α‐Motor neurons were visualized using anti‐NeuN antibody and anti‐mouse IgG1 Alexa Fluor488‐conjugated secondary antibody. C‐boutons and VGLUT1‐positive inputs were visualized using anti‐VAChT and anti‐VGLUT1 antibodies, respectively, and revealed using anti‐guinea pig or anti‐rabbit IgG Alexa Fluor555‐conjugated secondary antibody. α‐Motor neuron soma area was measured using ImageJ analysis software. Lipofuscin aggregates, defined as areas of lipofuscin in which the fluorescence from the cell body could not be seen through it, were also measured in ImageJ. Synaptic inputs were counted using ImageJ analysis software. For specifically counting C‐boutons and VGLUT1‐positive inputs on the soma, any puncta contacting the cell body of an α‐motor neuron were counted by hand. For counting the overall number of VAChT puncta in the ventral horn, the cell counter tool was used in the ImageJ software. The red (VAChT) color channel was separated, and a threshold of about 2% of the maximum was applied. Any puncta between 1 and 40 μm^2^ were counted. Anomalies were then removed by hand from the image with all channels visible using the ROI manager feature in ImageJ.

### 4.5 Imaging

All images were taken with a Zeiss LSM 700 or Zeiss LSM 710 confocal light microscope, with a 40× oil immersion objective (1.3 numerical aperture) using the Zeiss Zen Black software. α‐Motor neurons were identified by their size and location within the ventral horn of the spinal cord. Optical slices within the z‐stack were taken at 1.00‐μm intervals. Images were collapsed into a two‐dimensional maximum intensity projection for analysis.

### 4.6 Western blotting

Protein was isolated using NP‐40 lysis buffer and diluted in Laemmli buffer prior to denaturation at 95°C for 10 min. Proteins were separated on a 10% SDS‐PAGE gel. Membranes were blocked for 1 hr with 5% nonfat milk diluted in 0.1% Tween Tris buffer solution, incubated with primary antibody diluted in blocking solution overnight at 4°C, washed 3 times with 0.1% Tween Tris buffer solution, incubated in 1:10,000 HRP‐conjugated secondary antibodies for 4 hr at room temperature, and finally washed 3 more times with 0.1% Tween Tris buffer solution. Blots were visualized using Clarity™ Western ECL substrate (Bio‐Rad Cat# 170‐5060) and Chemidoc imager (Bio‐Rad). The primary antibodies and concentrations are displayed in Table 2.

### 4.7 Quantitative PCR

RNA was extracted from fresh‐frozen cervical mouse spinal cord using Trizol reagent (Life Technologies) and purified using the Aurum Total RNA Mini Kit (Bio‐Rad). RNA was reverse transcribed using iScript™ Reverse Transcription Supermix (Bio‐Rad). Quantitative PCR was performed with iTAQ SYBR Green Supermix (Bio‐Rad) containing using the CFX Connect Real‐Time PCR system (Bio‐Rad). Primer sequences are listed in Table 3.

**Table 3:** Primers used for Real‐Time PCR

Primer	Forward	Reverse
GAPDH	5′‐CCCACTCTTCCACCTTCGATG‐3′	5′‐GTCCACCACCCTGTTGCTGTAG‐3′
ChAT	5′‐CCTGGATGGTCCAGGCACT‐3′	5′‐GTCATACCAACGATTCGCTCC‐3′
HB9	5′‐CCAAGCGTTTTGAGGTGGC‐3′	5′‐GGAACCAAATCTTCACCTGAGTCT‐3′
ISL1	5′‐CGTGCTTTGTTAGGGATGGG‐3′	5′‐CATTTGATCCCGTACAACCTGAT‐3′
ISL2	5′‐CCGCAAGCTTTGCTCACATC‐3′	5′‐CCGGGCTTCTTCTTGGAATG‐3′
NeuN	5′‐GTGGCTGACTGTGCTGTTTGG‐3′	5′‐CACAGGCAGCTTTTCAACCTCT‐3′
VAChT	5′‐GAGAGTACTTTGCCTGGGAGGA‐3′	5′‐GGCCACAGTAAGACCTCCCTTG‐3′

### 4.8 Statistics

Significance between animals of different ages was determined using unpaired *t* test and one‐way ANOVA with post hoc Bonferroni test. The Kolmogorov–Smirnov test was used to compare the distribution of α‐motor neuron soma size between groups. Data are expressed as the mean ± *SE* (standard error), and *p *≤* *.05 was considered statistically significant.

## CONFLICT OF INTEREST

None.

## AUTHOR CONTRIBUTIONS

N.M. and R.C. performed all immunostaining, qPCR, and Western blotting assays; collected and analyzed data for each assay; and generated figures; N.S. collected and analyzed data; K.L.V., M.D.S., R.dC., and J.A.M. collected and provided spinal cords from rhesus monkeys; N.M., R.C., R.dC., KLV, and J.A.M. edited the manuscript; G.V. designed and supervised all experiments, and wrote the manuscript.
