# Supplementary figures and images for: α‐Motor neurons are spared from aging while their synaptic inputs degenerate in monkeys and mice
Source: Aging Cell. 2018 Feb 4;17(2):e12726. doi: 10.1111/acel.12726 (PMC5847869; doi:10.1111/acel.12726)

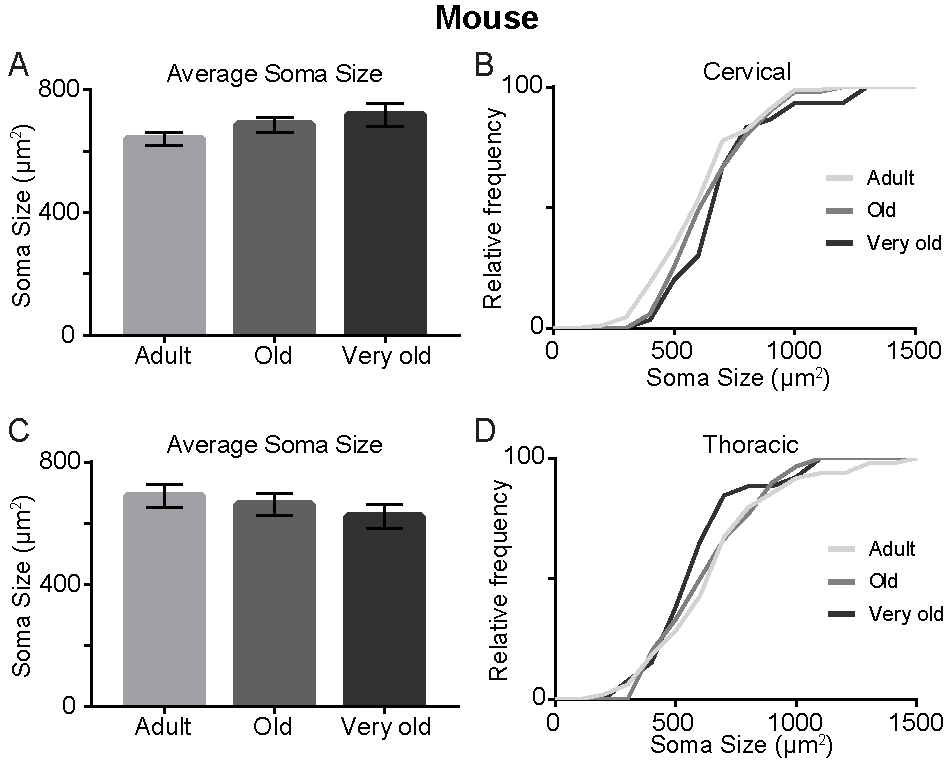

Supplement: Supplementary file 1 [file ACEL-17-e12726-s001.tif]

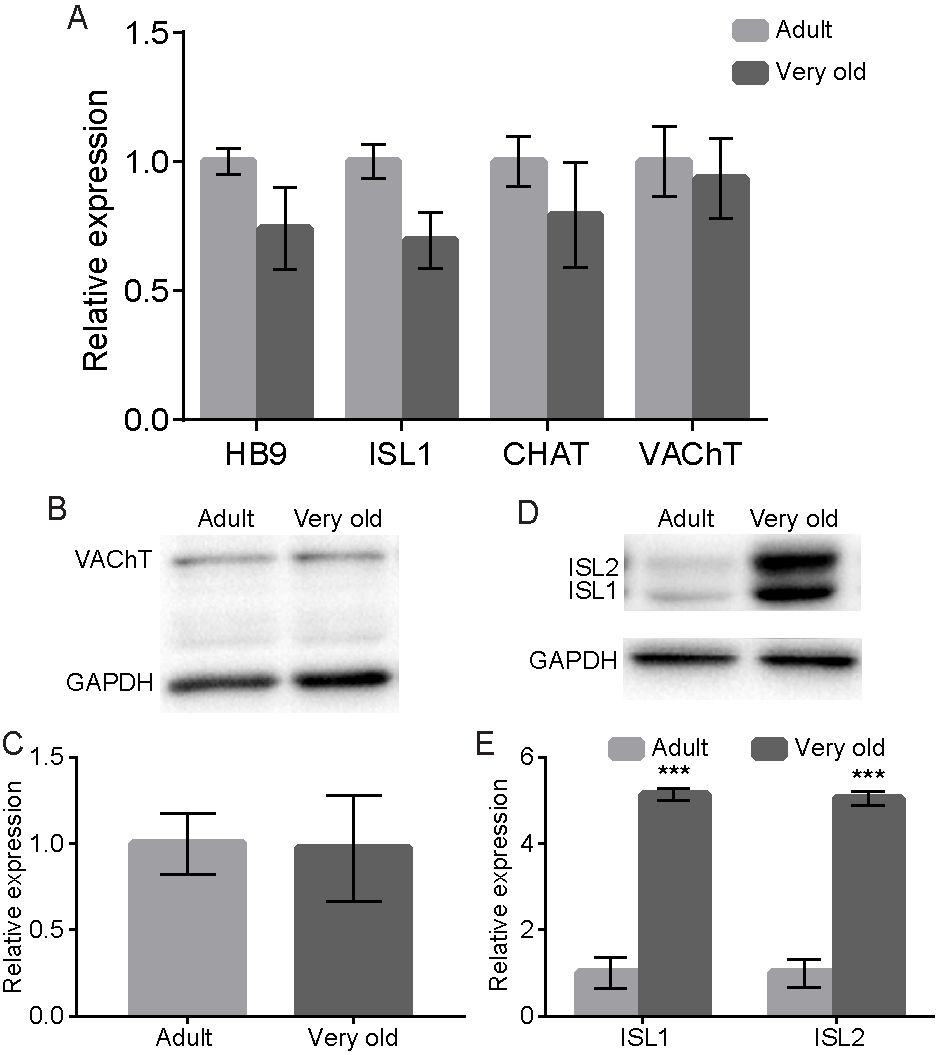

Supplement: Supplementary file 2 [file ACEL-17-e12726-s002.tif]

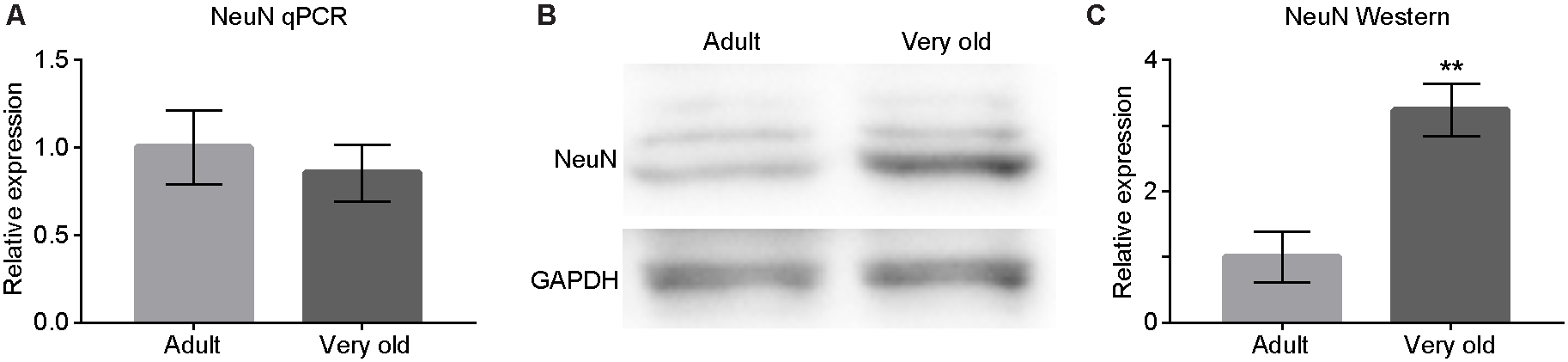

Supplement: Supplementary file 3 [file ACEL-17-e12726-s003.tif]

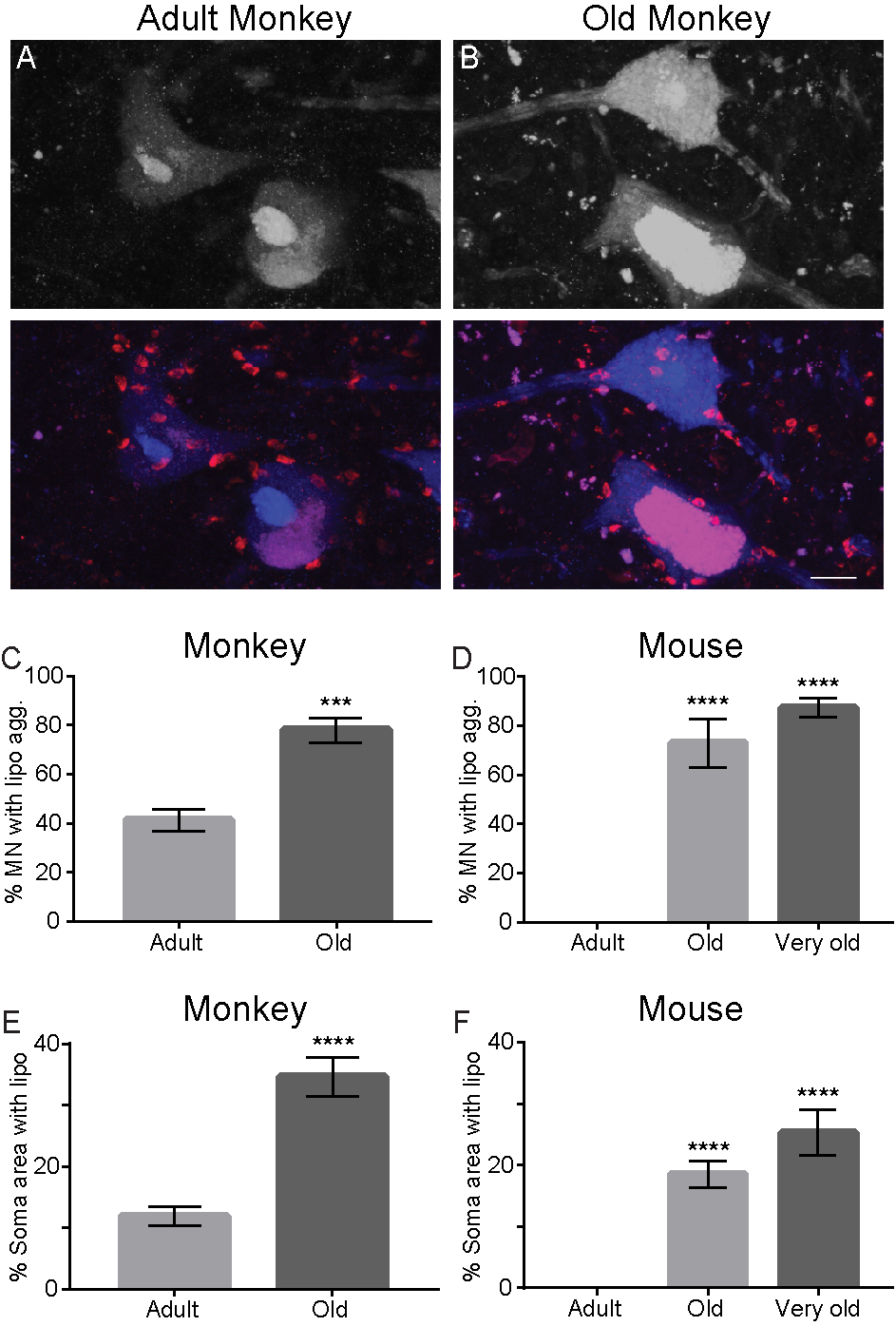

Supplement: Supplementary file 4 [file ACEL-17-e12726-s004.tif]

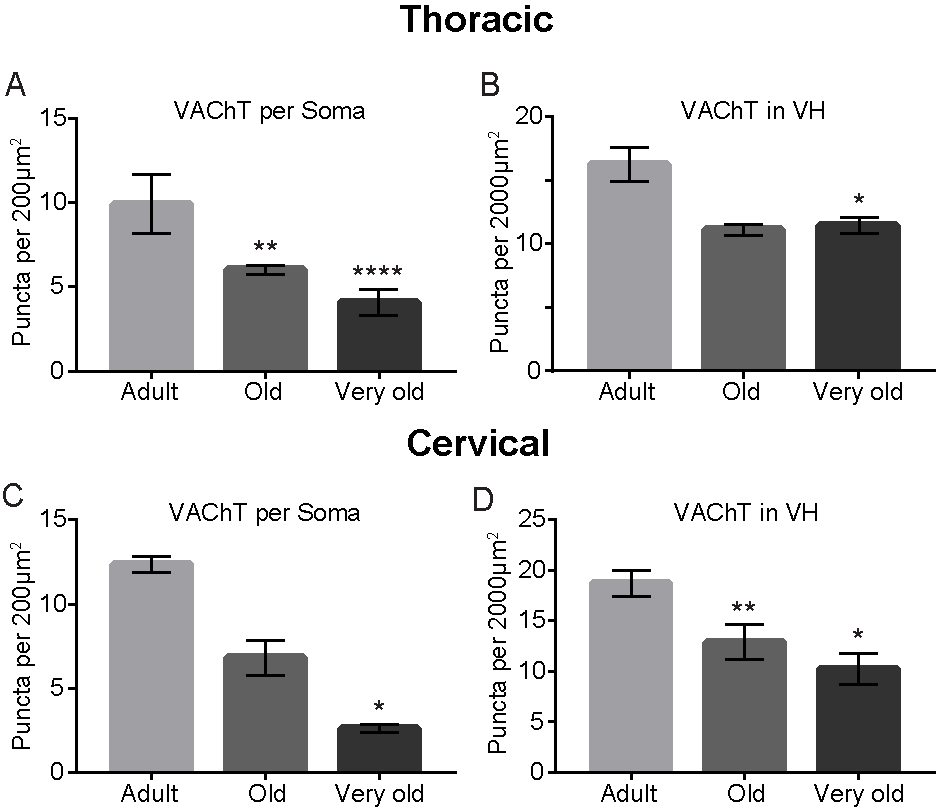

Supplement: Supplementary file 5 [file ACEL-17-e12726-s005.tif]

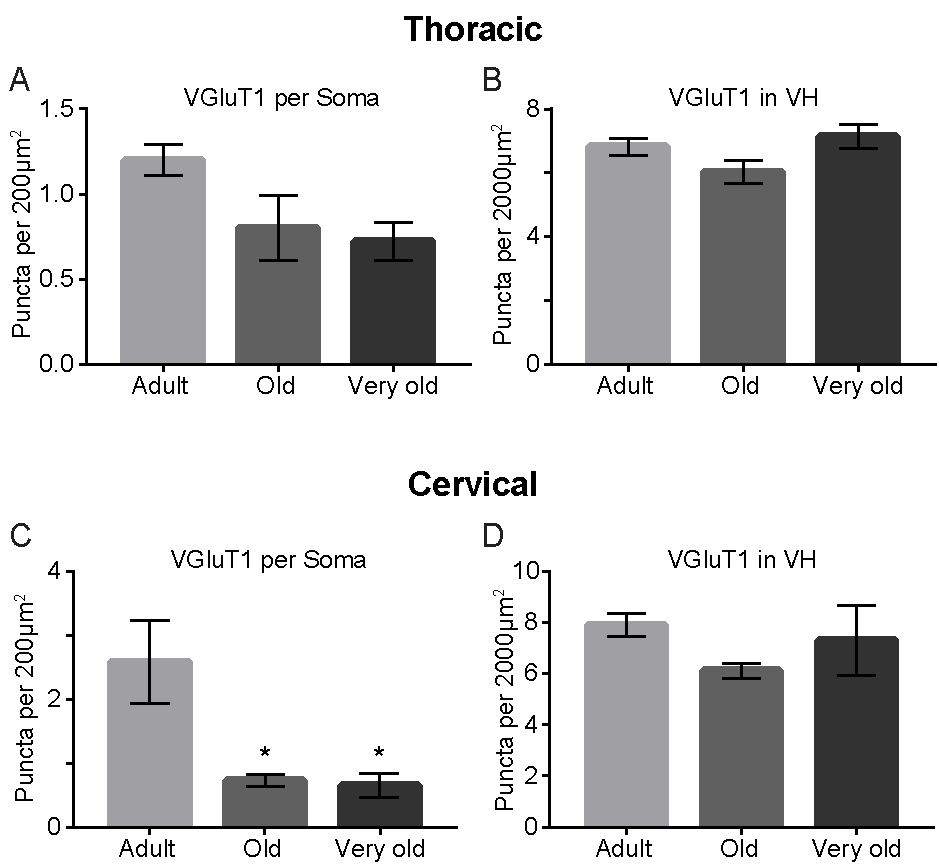

Supplement: Supplementary file 6 [file ACEL-17-e12726-s006.tif]

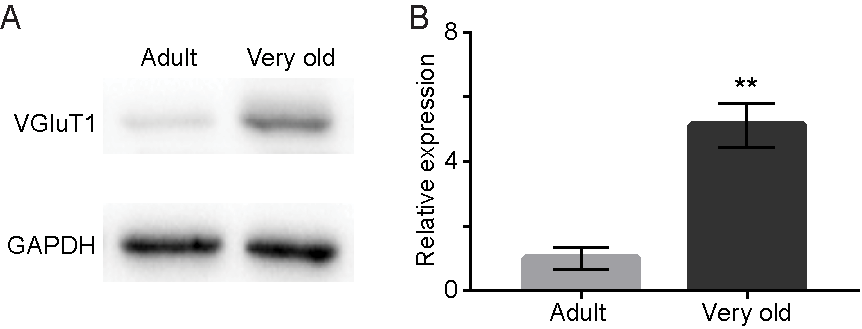

Supplement: Supplementary file 7 [file ACEL-17-e12726-s007.tif]
